# Canine Adipose Derived Mesenchymal Stem Cells Transcriptome Composition Alterations: A Step towards Standardizing Therapeutic

**DOI:** 10.1155/2017/4176292

**Published:** 2017-01-26

**Authors:** Nina Krešić, Ivana Šimić, Ivana Lojkić, Tomislav Bedeković

**Affiliations:** Virology Department, Croatian Veterinary Institute, Savska Cesta 143, 10,000 Zagreb, Croatia

## Abstract

Although canine adipose derived stem cells (cASCs) morphology characteristics and differentiation ability are well documented, transcriptome alterations of undifferentiated cASCs during ex vivo cultivation remain unknown. Here we demonstrate, for the first time, the transcriptome composition of isolated cASCs in undifferentiated state originating from six donors. Transcriptome changes were monitored during ex vivo cultivation between passage 3 (P3) and P5, which are mostly used in therapy. Influence of donors' age in given passage number on transcriptome composition was also investigated. Cultivation from P3 to P5 resulted in 16 differentially expressed genes with cooverexpression of pluripotency and self-renewal transcription factors genes SOX2 and POU5F1 dominant in old donors' cells. Furthermore, cASCs demonstrated upregulation of IL-6 in young and old donors' cells. In addition, ex vivo cultivation of cASCs revealed well-known morphological alterations accompanied with decrease in expression of CD90 and CD44 markers in P4 and higher monitored by flow cytometry and successful osteo- and chondrodifferentiation but inefficient adipodifferentiation in P3. Our results revealed the impact of ex vivo cultivation on nature of cells. Correlation of transcriptome changes with secretome composition is needed and its further impact on therapeutic potential of cASCs remains to be evaluated in clinical trials.

## 1. Introduction

Mesenchymal stem cells (MSCs), their unique immunoregulatory properties, and capacity for self-renewal, combined with multilineage differentiation, are some of the unique features that make them potent for therapeutic application [1, 2].

With mission to drive the translation of all cellular therapies for the benefit of patients International Society for Cellular Therapy (ISCT) established minimum criteria for definition of MSCs [3, 4]. However, the characterization of canine MSC is poorly defined compared to human MSC (hMSC) [5, 6]. Canines share many similar pathologies with humans; they represent perfect model for human conditions, much better than artificially created diseases in laboratory animals [7]. Thus, research on canine adipose derived mesenchymal stem cells (cASCs) may provide insight into stem cell therapy not just for canines but for humans as well.

To generate sufficient number of cASCs for therapeutic application, which are required in large proportions, long term culture is needed [8]. Simultaneously, in vitro expansion provokes continuous changes in the form of decayed proliferation rate, increased cell size, affected differentiation potential, acquired chromosomal instabilities, and molecular changes [9]. Although MSC populations become more homogeneous with serial passaging [8], inevitably gene expression changes (i.e., transcriptome changes) are occurring which might also have therapeutic consequences [9].

Driven by endeavor to light up cASCs properties exposed to harsh and intense conditions in cell culture flasks unlike “stem friendly niche” in residence tissue, present study for the first time brings cASCs transcriptome in time course. Here, we present new sight into peculiarities of cASCs regarding expansion, differentiation, and immunophenotype characteristics, with special attention to level of change in expression of 84 key genes, between passage 3 (P3) and P5, mostly used for therapy. Furthermore, we investigated whether donors' age affects baseline difference in gene expression.

Obtained results revealed peculiarities of culture-expanded cASCs, their transcriptome composition alterations which have potential to serve as valuable tool for prediction of cASC secretome. Better understanding of transcriptome composition during unavoidable stem cells cultivation may contribute to correlation of clinic outcome with therapeutic input.

## 2. Methods

### 2.1. Cultivation and Expansion of Canine Adipose Derived Stem Cells

#### 2.1.1. Adipose Tissue Collection

Abdominal adipose tissue samples from six female pet dogs (three young (8 months–2,7 years) and three aged (10–11,7 years)) of different breeds were selected for this study. All donors were dehelmintizated and vaccinated prophylactically against rabies, distemper, canine parvovirus, canine adenovirus 2, canine parainfluenza, and* Leptospira* spp. and referred to surgery. The adipose tissue of each dog was collected as medical waste.

#### 2.1.2. Isolation of cASCs

All collected samples were stored at 4°C and processed within 8 h after sampling. Isolation of the cASCs was performed using a minimum 5 g of abdominal adipose tissue. Samples (*n* = 6) were washed with sterile PBS (in house reagent) with addition of 1% antibiotic (penicillin/streptomycin, p/s, Sigma-Aldrich, USA), minced, and placed in 0,2% collagenase type I solution (ThermoFisher Scientific, USA) for digestion during 50 minutes at 37°C, 5% CO_2_, and 95% humidity, briefly stirring every 10 minutes. Foetal bovine serum (10%) (FBS, ThermoFisher Scientific, USA) was added to digested tissue; suspension was filtered through cell strainer 70 *μ*m (BD Bioscience, USA) and centrifuged (Hettich Rotina 420, Germany) 5 minutes at 2000 rpm (1400 ×g). Cell pellet was resuspended in 10 ml Dulbecco's Modified Eagle's Medium (DMEM) Low Glucose (ThermoFisher Scientific, USA) and centrifuged again at the same conditions. Finally, pellet was resuspended in prewarmed 79% DMEM Low Glucose + 20% FBS + 1% p/s (basal media) and incubated at 37°C, 5% CO_2_, 95% humidity. The media were changed 24 h later and all nonadherent cells were removed. Confluent, adherent cells were designated P0. Passaging was performed in T75 cell culture flask (Nunc, ThermoFisher Scientific, USA) using basal media. Cells were cryopreservated in P2 in 90% FBS + 10% DMSO (Sigma-Aldrich, USA) at −80°C using Nalgene Cryo −1°C Freezing Container and then placed in liquid nitrogen. After thawing cells were cultivated in T25 cell culture flasks (Nunc, Thermo Scientific, USA). Passaging was performed at confluence of 80% up to P7. The cASCs suspensions were culture negative for bacteria and fungi and polymerase chain reaction (PCR) was negative for* Mycoplasma* spp.

### 2.2. Differentiation Assay

To evaluate the “stemness” of established cultures, cells in P3 after cryopreservation were induced to differentiate toward trilineage (adipogenic, osteogenic, and chondrogenic). Protocols were performed according to manufacturer's instructions (Miltenyi Biotec, Germany) with modifications. All trilineage differentiation tests were performed using 96-microwell plate (Nunc, Thermo Scientific, USA) by seeding 2.0 × 10^4^ cells per well in basal media. After 48 h it was decanted and 300 *μ*l StemMACS AdipoDiff. Media for adipocytes, StemMACS OsteDiff. Media for osteoblasts, and StemMACS ChondroDiff. Media for chondrocytes (Miltenyi Biotec, Germany) were added to particular wells except for control wells which were further cultivated in basal media. Differentiation and basal media were changed every 48–72 hours and plates were microscopically (Zeiss, Germany) examined (×10). Differentiation was performed during 15 days.

#### 2.2.1. Detection of Adipocytes 

Detection of adipocytes was performed by removing StemMACS AdipoDiff Media and washing the cells twice with 300 *μ*l of sterile PBS (in house reagent). Cells were fixed with 300 *μ*l of methanol (Carlo Erba, France) and incubated 5 minutes at room temperature (RT). Methanol was aspirated completely, cells were washed twice with deionized H_2_O, and 300 *μ*l of Oil Red O (Sigma-Aldrich, USA) was added to all wells. Plates were incubated 20 minutes at RT. Oil Red O was aspirated, cells were washed 2x with deionized H_2_O, and finally 100 *μ*l of deionized H_2_O was added to keep cells moisture. Immediately after staining cells were examined under microscope and pictures were taken with camera (Zeiss, Germany). Red color stained cells were considered to be positive.

#### 2.2.2. Detection of Osteoblasts

Detection of osteoblasts was performed by removing StemMACS OsteoDiff. Media (Miltenyi Biotec, Germany) and washing the cells twice with 300 *μ*l of sterile PBS (in house reagent). Cells were fixed by adding 300 *μ*l of ice cold methanol (Carlo Erba, France) and incubating 5 minutes at −20°C. Methanol was aspirated completely, cells were washed twice with deionisated H_2_O, and 300 *μ*l of SIGMA*FAST* BCIP/NBT substrate (Sigma-Aldrich, USA) was added to all wells. Plates were incubated 10 minutes at RT. Substrate was aspirated, cells were washed 2x with deionisated H_2_O, and finally 100 *μ*l of deionisated H_2_O was added to keep cells moisture. Immediately after staining stained cells were examined under microscope and pictures were taken with camera. Purple stained cells were considered to be positive.

#### 2.2.3. Detection of Chondrocytes

Detection of chondrocytes was performed by carefully removing StemMACS ChondroDiff. Media (Miltenyi Biotec, Germany) without aspirating spheroids and washing the spheroids twice with 300 *μ*l of sterile PBS (in house reagent). Spheroides were fixed by adding 300 *μ*l of neutral buffered formalin (10%) (in house reagent) and incubating 60 minutes at RT. Formalin was aspirated and spheroids were washed twice with deionisated H_2_O. Alcian staining solution (60 ml 98–100% ethanol + 40 ml acetic acid (98–100%) + 10 mg Alcian Blue 8 GX (Sigma-Aldrich, Germany)) (300 *μ*l) was added to carefully cover spheroids and incubated overnight at RT in the dark. Alcian staining solution was removed and spheroids were washed with destaining solution (120 ml 98–100% ethanol + 80 ml acetic acid (98–100%)) 2x for 20 minutes. Destaining solution was aspirated and 300 *μ*l PBS was added. Immediately after staining cartilage spheroids were observed for intensive dark-blue. Spheroids were carefully transferred to microscopic slides and pressed with cover slide. Preparations were microscopically examined and pictures were taken with camera.

### 2.3. Senescent Cells Detection

Detection of senescent cells was performed using Senescence Detection Kit (Abcam, UK) which is designed to histochemically detect senescence associated (SA), beta-Gal activity in culture cells. The SA-beta-Gal is present only in senescent cells and is not found in presenescent, quiescent, or immortal cells. Cells in passages 4, 5, and 6 were seeded in 12-well plates. In this assay, the monolayered cells cultured in a 12-well plate (5 × 10^4^) overnight were washed in phosphate-buffered saline (PBS, pH 7.4) and then fixed for 15 min with 0,5 ml of fixative solution at room temperature. After washing in PBS, the fixed cells were incubated in the staining solutions mix (staining solution, staining supplement, and 20 mg/ml X-Gal in DMSO) with incubator at 37°C over night without supplying CO_2_. The cells were then examined under a stereomicroscope (Stereo Discovery, V20, CL1500 ECO, Zeiss) at 18x and 60x magnification for qualitative detection of SA-beta-Gal activity.

### 2.4. Cell Characterization by Flow Cytometry

All samples were measured on six color, two laser FACSVerse (BD Biosciences, USA), serial number Z6511540253. Target channels were defined for all fluorochromes of settings using calibration bead particles (BD FACSuite CS&T Research Beads). Cells were analyzed in P1–P6, respectively. Media were decanted; cells were washed with 10 ml DMEM Low Glucose (ThermoFisher Scientific, USA) and detached with 3,3 ml Accutase (eBioscience, USA). Cell Wash (BD Biosciences, USA) with 20% FBS was added for resuspension; cells were counted and split into 4 tubes (BD Biosciences, USA) each containing 1 × 10^5^ cells/mL and centrifuged (235 ×g 5 min). Supernatant was decanted and pellets were briefly finger tapped. Staining was performed using commercially available fluorochrome-conjugated anti-canine monoclonal antibodies (Table 1) during 30 minutes at 4°C in the dark, washed with Cell Wash (BD Biosciences, USA), and centrifuged 5 minutes at 235 ×g. Finally, cells were resuspended in 500 *μ*l of Cell Wash for flow cytometric analysis.

Experimental settings were set up using unstained cells, single stain, appropriate isotype controls (Table 1), and FMO controls to establish the boundary between negative and positive fluorescent regions. Further experiments were performed using unstained cells, mixture of isotype controls, and mixture of mentioned CDs and FMO for APC. Exclusion of nonviable cells (0-1%) was performed using Propidium Iodide staining solution (BD Bioscience, USA) for all analyzed cell samples. Compensation has been performed automatically. The same gating strategy has been used for all data files. The results for 10,000 acquired events were expressed as the percentage of cells falling above the negative region for MSCs positive markers. Results were analyzed using FACSuite software.

### 2.5. Gene Expression Analysis

For the gene expression analysis real time PCR array method was chosen. To analyze genes expressed in isolated and culture-expanded undifferentiated cASCs (Figure 4) commercially available validated RT^2^ Profiler PCR Array Format R suitable for use with Rotor-Gene Q (Qiagen, Germany) was used. Array detects the expression of 84 genes classified into four major groups of markers (stemness, MSC specific, associated with MSC, and MSC differentiation). This array includes SYBR green-optimized primer assays.

Change in level of relative gene expression between P3 and P5 was assessed. The underlying criterion for choosing the mentioned passages was microscopically identified alterations in phenotype of cultured cASCs as well as use of cells in these passages in therapeutic purposes.

Total RNA was isolated using RNeasy Mini Kit (Qiagen, Germany) following manufacturer's instructions. Integrity of RNA was examined by 1% agarose gel electrophoresis; the RNA concentration and purity were determined by measuring the absorbance in a Nanophotometer P360 (Implen, Germany). Obtained results matched the criteria needed for downstream application of RNA prescribed by array manufacturer (A_260_ : A_230_ ratio greater than 1,7; A_260_ : A_230_ ratio 1,8–2,0; concentration determinate by A_260_ > 40 *μ*g/ml). RT^2^ First Strand Kit (Qiagen, Germany) was used for genomic DNA elimination and cDNA synthesis which served as template for RT^2^ Profiler PCR Array (Qiagen, Germany).

Cycling conditions and the starting amount of RNA were used according to manufacturer's instructions. Obtained data were analyzed using RT^2^ Profiler PCR Array Data Analysis version 3.5 software available at http://pcrdataanalysis.sabiosciences.com/pcr/arrayanalysis.php?target=upload. The software analyzes the data using ΔΔCt method and performs statistical analysis of the data (based on Student's *t*-test); differences between gene expression levels were considered significant when *p* < .05. Fold change cutoff of 2 was chosen.

To monitor expression changes through passages and considering donor age we created the following comparison set (CS):

(CS1) P3 (6 donors) (control group) versus P5 (6 donors) (test group)
(CS2) P3 of young donors' cells (*n* = 3) (control group) versus P5 of young donors' cells (*n* = 3) (test group)
(CS3) P3 of old donors' cells (*n* = 3) (control group) versus P5 of old donors' cells (*n* = 3) (test group)
(CS4) P3 of young donors' cells (*n* = 3) (control group) versus P3 of old donors' cells (*n* = 3) (test group)
(CS5) P5 of young donors' cells (*n* = 3) (control group) versus P5 of old donors' cells (*n* = 3) (test group)

(CS1) presents the changes in relative expression levels of 84 genes (Figure 3) of cASCs from 6 donors between P3 and P5. (CS1) is the most representative one since it includes six biological replicates in each control and test group. (CS2) and (CS3) are created by dividing donors in age groups: young (*n* = 3) and old (*n* = 3) to monitor transcriptome changes during cultivation. (CS4) and (CS5) are made to compare levels of expression in young and old donors' cells in the same passage.

#### 2.5.1. Gene Filtering

Gene filtering was performed by selecting genes with stable changes in expression (threshold cycle (Ct) value < 30) for further analysis and excluding genes with relatively low or nondetected expression either in test or control group (average Ct > 30 for one group and average Ct < 30 for another group) as suggested by software instructions. Although aware of possible biological importance of those excluded genes, it would be dangerous to interpret those data without using more biological replicates for verification of those results.

Obtained results were further analyzed using Gene Ontology.

### 2.6. Statistica

Further statistical analysis of results obtained for CSs was performed using software Stata 13 using Fisher's exact test and *p* value ≤ .05 was considered significant.

## 3. Results

### 3.1. Expansion and Differentiation Characteristics

**Figure 1:**
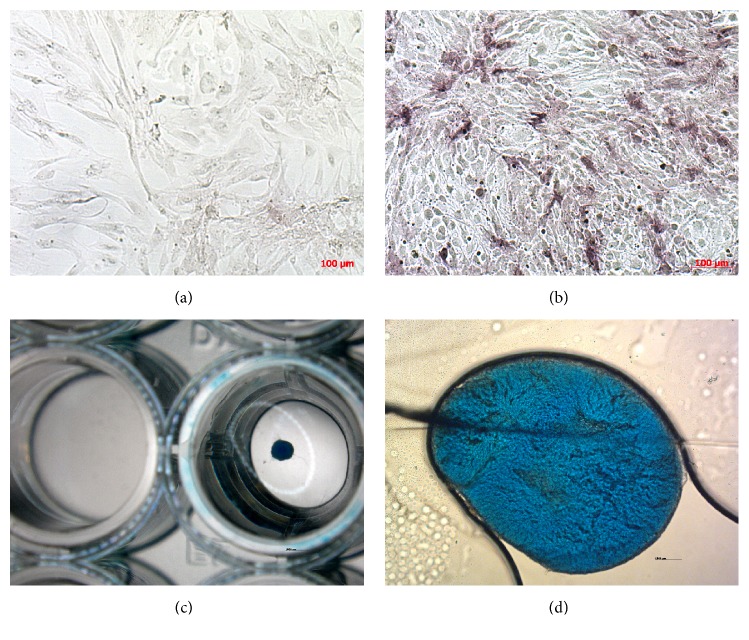
Differentiation of canine adipose derived stem cells. (a) Canine adipose derived stem cells negative for alkaline phosphatase activity (negative control for osteoblasts differentiation). (b) Osteoblast differentiation. Canine adipose derived stem cells stained for alkaline phosphatase activity with NBT substrate (purple). (c) Chondrodifferentiation of canine adipose derived stem cells in microwell plate stained with Alcian Staining Solution, negative and positive well. Positive well contains blue spheroid. (d) Chondrodifferentiation of canine adipose derived stem cells. Spheroid stained with Alcian Staining Solution.

Isolated adherent fibroblast-like cASCs during expansion demonstrated enlargement already in P3, morphological alteration in P4 (cuboidal shape, roundness, and plate appearance were present in part of cells in flask), and ultimately proliferation arrest in P7 regardless of age of donor. Expansion characteristics observed microscopically were similar in all donors. Canine ASCs were refractory to adipogenic differentiation but able to differentiate into osteoblasts (Figure 1(b)) and chondrocytes (Figures 1(c) and 1(d)).

### 3.2. Detection of Senescent Cells

**Figure 2:**
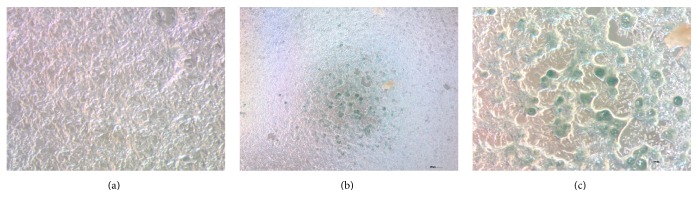
Detection of senescent cells. Qualitative SA-*β*-Gal assay of the most representative donors' canine adipose derived stem cells in passage 4. A local region of senescence cell is shown (b, c). Negative control cells (a).

SA-beta-Gal activity was detected already in P4 in minority of cells in all donors (blue staining) (Figure 2). With each passage (5, 6) higher number of cells showed marked SA beta-Gal activity.

### 3.3. Flow Cytometry Surface Marker Profile

**Figure 3:**
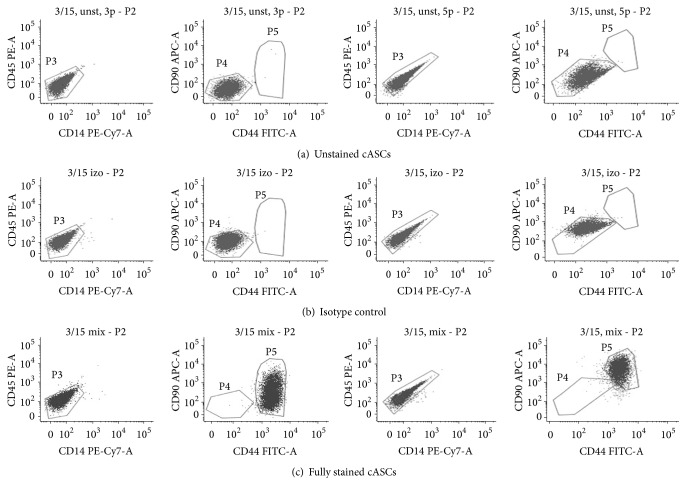
Flow cytometry results. The most representative canine adipose derived stem cells from one donor are shown in passage 3 and 5. The figure shows P2 population after excluding dead cells and doublets. CD: cluster of differentiation, APC: allophycocyanin, PE: Phycoerythrin, FITC: fluorescein isothiocyanate, and PE-Cy7: R-Phycoerythrin-Cyanine 7.

**Figure 4:**
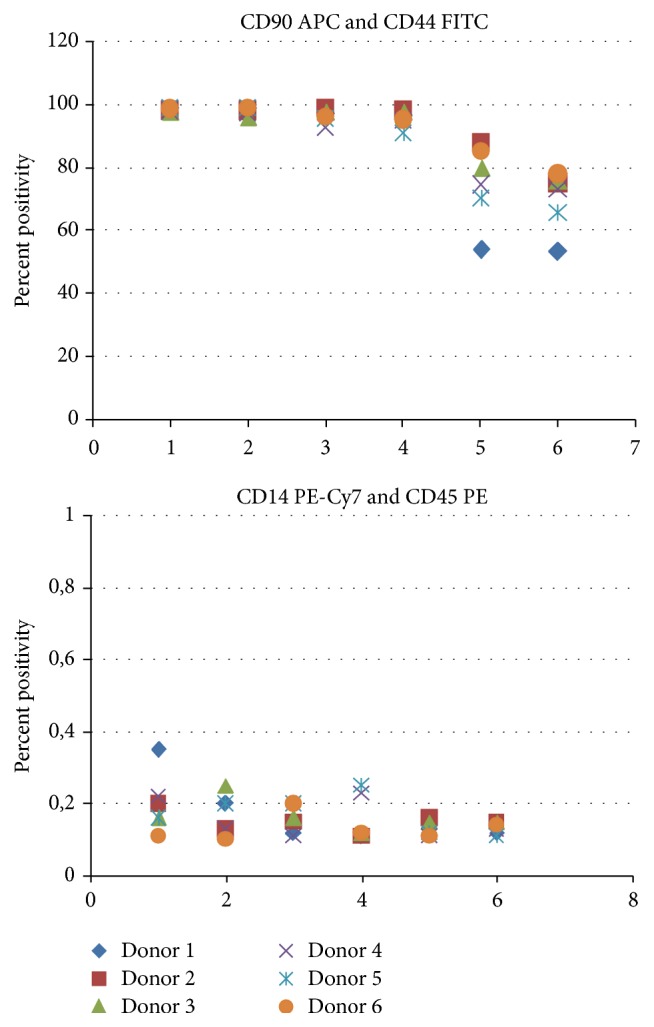
Flow cytometry results. Expression of cluster of differentiation (CD) during cultivation from passage 1 (P1) to P7.

Undifferentiated canine ASCs were analyzed by flow cytometry (Table 1) to monitor the surface marker profile during their ex vivo expansion. Each passage demonstrated a homogeneous population of viable cells, with continuous expression of CD90, CD44, CD29 (>95% cells in population), and CD271 and the absence of CD45 and CD14 (Figures 3 and 4) cell markers from P1 to P4 irrespective of the donor included in the study. Small proportion of cells expressed CD271 (up to 3,21%). Canine ASCs expressed low levels of autofluorescency in P1-P2 and it started to increase in P3 to the end of cultivation which was accompanied by increased median of the forward and side scatter signal (Figure 2). However, expression of CD90 and CD44 decreased in P5-P6 in all donors cells (Figure 4).

Examined clusters of differentiation together with stem cell specific genes for CD105, CD73, and CD166 were also found at mRNA level in P3 and P5 using array. The examined markers showed stable expression level during that expansion period.

### 3.4. Gene Expression

**Figure 5:**
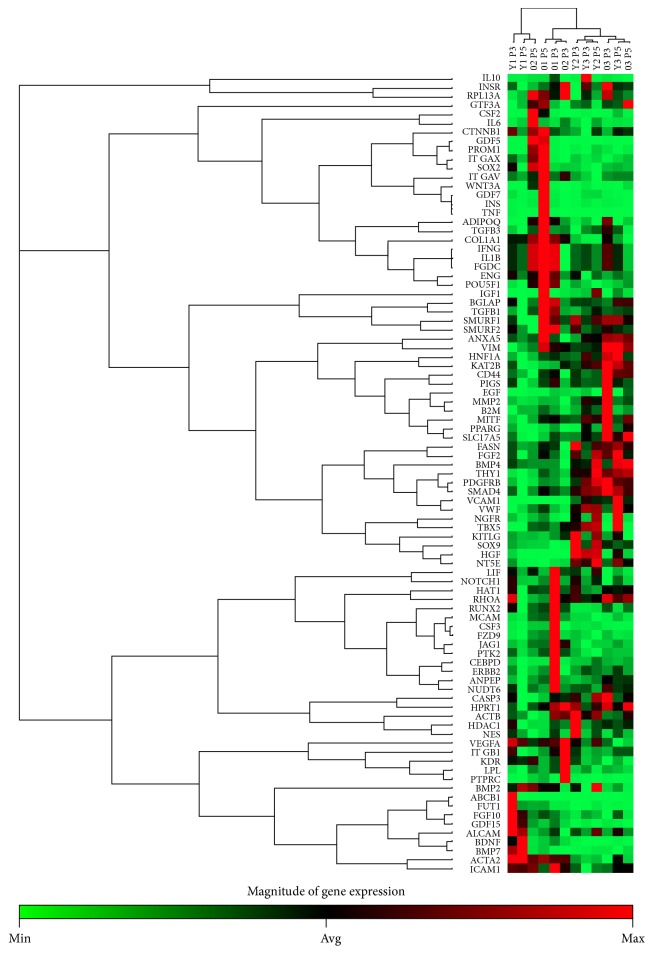
Clustergram presents 84 analyzed genes of cASCs of 6 donors (3 young donors (Y1, Y2, and Y3) in passage 3 (P3) and P5 and 3 old donors (O1, O2, and O3) in P3 and P5). Passage 3 of cASCs was used as control group and P5 of cASCs represents test group. Nonsupervised hierarchical clustering of the entire dataset displayed no age or passage number related clustering.

**Figure 6:**
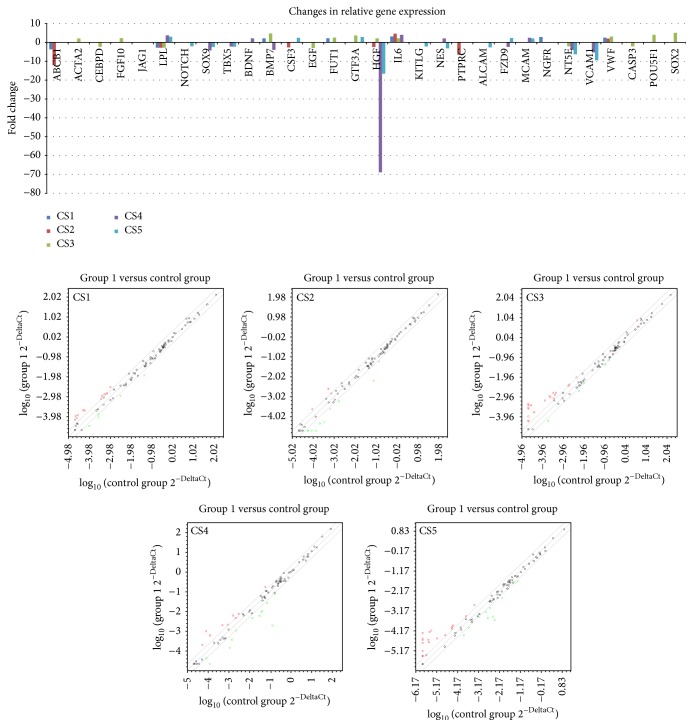
Graphical representation of gene expression results. Genes differentially expressed between passage 3 (P3) to P5 in each created comparison set (CS1–CS5) and their fold regulation values are indicated in chart. Scatter plot for 84 tested genes of canine adipose derived mesenchymal stem cells in each created comparison set (CS1–CS5).

Results (Table 2) were analyzed according to created comparison sets (CS) (Figures 5 and 6) as described.

Obtained data revealed in total 7 differently expressed genes in CS1. Comparing CS2 and CS3 it can be noted that young donors' cells in P5 demonstrated more downregulated genes (71,43%) than old donors' cells in P5 (33,34%) while results for upregulated genes are opposite (66,66% for old and 28,57% for young donors' cells). Difference between CS2 and CS3 was statistically significant (*p* ≤ .05). Comparing baseline difference in gene expression in P3 and P5 in donors of different age (CS4 and CS5) result was not statistically significant.

Stemness genes (bFGF, INS, LIF, POU5F1, and SOX2) were expressed at similar levels in young and old donors, except for CS3 where old donors' cells in P3 expressed higher levels of SOX2 and POU5F1.

To better understand cASC properties in biological and molecular context we analyzed obtained data using Gene Ontology. According to biological function changed genes were classified into those responsible for apoptotic process, biological adhesion, biological regulation, cellular process, developmental process, immune system, localization, metabolic process, reproduction, and response to stimulus. Their molecular function is related to binding, catalytic, and receptor activity.

## 4. Discussion

Although the “gold rush” in using mesenchymal stem cells (MSCs) for therapeutic purposes began with high enthusiasm numerous scientific issues remain to be resolved. Available literature offers a lot of information but still makes readership pretty confused (no uniform characterization criteria for MSCs [10], little consensus about immunomodulation of MSCs, secretome composition following infusion which is uncertain [11], and MSCs which are not always immunosuppressive [12]).

In stem cell transplantation, canines have been used for more than 30 years. As a model they are more comparable with humans than rodents. Clinical application of stem cells often means transplantation of cells with unknown characteristics [13]. It is important to identify the factors that are involved in the regulation of the expression and production of paracrine molecules in MSCs to achieve an optimal therapeutic outcome [14]. Therefore, this study provides comprehensive insight into cASCs from the aspects of morphology, differentiation, and immunophenotyping with special emphasis on transcriptome.

Morphological changes of cASCs in this study are in line with those described in the literature [15, 16]. However, senescent appearance, confirmed with SA-beta-Gal activity, was observed in P4 in part of flask seeded cells and P7 represents the upper cultivation limit. Those findings can be a result of culturing conditions. When cells are explanted from an organism and placed in culture, they have to adapt to abnormal concentrations of nutrients and growth factors, as well as the absence of surrounding cells and extracellular matrix [16]. Early senescence in this case could be due to high seeding density (3 × 10^4^/cm^2^) and high percentage of FBS (20%) in basal media. Limited lifespan (up to only P7) is confirmed continuously on multiple donors (*n* = 18, unpublished data) of different age and breeds in our laboratory conditions. Contributing factors to the limited lifespan are the same as those responsible for senescence, but generally shorter lifespan of canine species when compared to humans should be also considered. Dogs lose telomeric DNA approximately 10-fold faster than humans which is similar to the ratio of average life spans between these species [17]. Elongation of life span can be achieved by culturing in serum-free medium supplemented with a number of defined growth factors [18] or by culturing under physiological oxygen conditions [19] as described for mouse embryonic fibroblasts. Morphological and immunophenotypic cells obtained from all donors were similar which is in line with observations made by others [20].

For the differentiation test we have chosen human differentiation media because of their controlled origin and quality and to ensure equal conditions for all steps of differentiation. Lack of adipogenic differentiation in cASCs was probably due to applying human media as described by Neupane et al. who suggest usage of canine adjusted media [21]. Same authors also reported refraction of cells to osteogenic differentiation, but our results suggest the opposite. Differentiation results were further supported by upregulated BMP7 and IL-6 and downregulated LPL mRNA. The BMP signal induces osteoblastic differentiation, at the same time inhibiting adipogenesis and myogenesis [22]. LPL expression may reflect the growth-arrest stage which is prerequisite for adipocytes differentiation [23]. Upregulated BMP7 and downregulated LPL mRNA strongly support cASCs behavior during differentiation process, absence of adipogenic and successful osteogenic differentiation. Of further interest is the fact that pretreatment of MSCs with IL-6 inhibits adipogenic and chondrogenic differentiation [24]. Our results establish the need to investigate what is the role of endogenous IL-6 on inhibition of adipogenesis and influence on chondrogenesis.

For a more in-deep characterization the immunophenotyping was performed with flow cytometry. We have observed stable expression of analyzed markers during cultivation time which is in compliance with previous reports [25] and no differences in marker expression were observed between young and old donors. We also examined the expression of CD271 which has been proposed as marker of primary choice for tissue regeneration [26]. Expression values for CD271 in cASCs were similar as in human ASCs [27]. To complement this marker analysis based on detection using mAbs, which are often commercially unavailable, we also characterized cASCs at the mRNA level. Results of molecular analysis enabled us to see what genes those cells are expressing and which of them are changed during culturing from P3 to P5 and to what magnitude. Molecular analysis provides explanation and confirmation of the observed results (lack of adipogenic differentiation, immunophenotype properties, and stemness preservation). Therefore, these molecular techniques are valuable tool for fast screening of cells prior to application and for better understanding of therapeutic power of cASCs.

By analyzing results of created comparison sets (CS) using Gene Ontology one can see that part of changed genes in CS1 was involved in fundamental cellular functions but our attention was dragged by upregulated interleukin-6 (IL-6) as controversial molecule. IL-6 expressed continuous upregulation in CS 1, 2, 3, 4 with fold regulation values higher in young (CS2) than in old donors (CS3). This may indicate that young donors' cells are more potent promoters of immunomodulation. It is well-known fact that MSCs produce IL-6 whose biology is complex [28]. IL-6 is thought to be harmful because it can be key to the maintenance of chronic inflammation [29] but at the same time it stimulates the secretion of anti-inflammatory IL-10 [12]. Through production of IL-6, MSCs prevent the differentiation of monocytes towards antigen-presenting cells and skew differentiation towards an anti-inflammatory IL-10-producing cell type [30]. IL-10 was not produced by cASCs in present study which is in line with results of mentioned authors [30] who report IL-10 production exclusively by monocytes after exposure to MSCs-produced IL-6. cASCs following infusion meet microenvironment for interaction with immune cells and by IL-6 they could stimulate secretion of favorable IL-10. Based on the above-mentioned facts, we believe that upregulated IL-6 in cASC during culturing may indicate beneficial therapeutic effect. However, it should be noted that IL-6 and IL-10 are not the only cytokines involved in these complex interactions. Paracrine mechanisms for the therapeutic effects of MSC are very complex and involve large number of growth factors, cytokines, signaling molecules, and related receptors with a broad range of biological functions which should be investigated in future.

Comparison of CS2 and CS3 enabled us to investigate influence of cASC cultivation on changes in relative gene expression in different age groups. Interestingly, old donors' cells (CS3) exhibited 2,14-fold higher changed genes, mostly upregulated, which could indicate more intense processes within those cells as response to cultivation conditions. Only two genes were in common, upregulated IL-6 and downregulated LPL. Fold regulation of IL-6 was 2,13-fold higher in young donors' cells and fold regulation for LPL was the same in both CS. Only old donors' cells expressed upregulation of two stemness markers, SOX2 and POU5F1. These transcription factors for pluripotency and self-renewal are naturally expressed in MSCs at low levels in early passages and gradually decrease as the passage number increases [31]. The effect of cooverexpression of POU5F1 and SOX2 in human adipose tissue MSCs (hAT-MSC) has been investigated. Those results show effectively enhanced mesodermal differentiation potency indicating increased stemness of hAT-MSCs [31]. Seen upregulation of these genes speaks for preserved stemness characteristics of old donors' cells which raises hope from therapeutic perspective, but their involvement in tumor genesis should always be kept in mind. It remains to view what characteristic will be predominant in older hosts since incidence of tumor formation increases with age [32, 33] and aging represents the single biggest risk factor for most cancers [34–36]. Furthermore, genetically unmodified MSCs can undergo chromosomal abnormalities even at early passages and form malignant tumors when transplanted in vivo. Careful monitoring of chromosomal status is warranted when in vitro expanded MSCs are used for cell therapy [37].

Comparison of CS4 and CS5 enabled us to investigate differences in transcriptome of cASC in the same passage but different donors' age group. Old donors' cells in lower (P3) (CS4) and higher passage (P5) (CS5) differently expressed genes mainly differentiation associated and downregulated. This together with upregulated stemness genes in CS3 speaks for preserved undifferentiated state what is important for therapy. How exactly donors' age affects level of change in gene expression of cASCs has to be further investigated in future on higher number of biological replicates. Beside age, gender, environment, and passage number, anatomical site of tissue harvesting should be taken into account when analyzing changes in relative gene expression. Although mRNA might be useful in predicting protein expression (i.e., secretome), everything done has to be confirmed at protein level.

Differentiation results are in line with array results which speak for its usefulness as fast screening method. Beside the fact that SOX2 and POU5F1 indicate increased stemness of cASCs, involvement of those genes in tumor genesis raises questions about autologus stem cell therapy in elderly individuals. The role of the endogenous IL-6 of cASCs following transplantation has to be evaluated for diverse clinical conditions because of its context-dependent pro- and anti-inflammatory properties. Transcriptomic results revealed the need to monitor and determine the potential influence of SOX2, POU5F1, IL-6, and other molecules as cASCs secretome components in the stem cell therapy.

## Figures and Tables

**Table 1:** Antibody panel used for flow cytometry analysis.

Cell surface marker	Antibody clone	Species reactivity	Host	Clonality	Source	Expression on cASCs
*CD14 PE-Cy7*	M5E2	Canine, human	Mouse	Monoclonal	BD Pharmingen	Negative marker
*CD44 FITC*	YKIX337.8	Canine	Canine	Monoclonal	eBioscience	Positive marker
*CD45 PE*	YKIX716.13	Canine	Rat	Monoclonal	AbD Serotec	Negative marker
*CD90 APC*	YKIX337.217	Canine	Canine	Monoclonal	eBioscience	Positive marker
*CD271 FITC*	ME20.4-1.H4	Canine	Mouse	Monoclonal	Miltenyi Biotec	Positive marker
*CD 29 FITC*	MEM-101A	Human, canine, pig	Mouse	Monoclonal	Antibodies-online.com	Positive marker
IgG2a PE-Cy7	G155–178		Mouse	Monoclonal	BD Pharmingen	
IgG2a FITC	eBR2a		Rat	Monoclonal	eBioscience	
IgG2b PE	R35–38		Rat	Monoclonal	BD Pharmingen	
IgG2b APC	eB149/10H5		Rat	Monoclonal	eBioscience	

CD: cluster of differentiation, Ig: immunoglobulin, APC: allophycocyanin, PE: Phycoerythrin, FITC: fluorescein isothiocyanate, PE-Cy7: R-Phycoerythrin-Cyanine 7.

**Table 2:** Results of relative gene expression analysis. Summary of all results in different comparison set (CS).

GENE NAME	CS1	CS2	CS3	CS4	CS5
	All donors	Y P3/Y P5	O P3/O P5	Y P3/O P3	Y P5/O P5
ABCB1	**−3,70↓**	**−12,09↓**		**−2,70↓**	*3,16*↑
ACTA2			*2,09*↑		
CEBPD			**−2,32↓**		
FGF10			*2,23*↑	**−3,05↓**	**−2,50↓**
JAG1				*2,93*↑	
LPL	**−2,82↓**	**−2,82↓**	**−2,82↓**	*3,75*↑	*3,02*↑
NOTCH					**−2,05↓**
SOX9				**−4,24↓**	**−2,45↓**
TBX5				**−2,19↓**	**−2,3↓**
BDNF				*2,10*↑	
BMP7	*2,10*↑		*4,76*↑	**−3,98↓**	
CSF3		**−2,65↓**			*2,38 * **↑**
EGF			**−2,98↓**		
FUT1	*2,15*↑		*2,55*↑		
GTF3A			*3,68*↑		*2,84*↑
HGF		**−2,44↓**	*2,10*↑	**−68,91↓**	**−16,6↓**
IL6	*3,16*↑	*4,59*↑	*2,15*↑	*4*↑	
KITLG					**−2,18↓**
NES				*2,09*↑	**−3,2↓**
PTPRC		**−6,52↓**			
ALCAM					**−2,65↓**
FZD9				**−2,36↓**	*2,33*↑
MCAM				*2,42*↑	*2,08*↑
NGFR	*2,81*↑				
NT5E			**−2,06↓**	**−3,98↓**	**−6,29↓**
VCAM1				**−5,37↓**	**−9,5↓**
VWF	*2,48*↑	*2,00*↑	*3,08*↑		
CASP3			**−2,11↓**		
POU5F1			*3,97*↑		
SOX2			*5,07*↑		
Total (%)	7 (100)	7 (100)	15 (100)	15 (100)	16 (100)
Upregulated (%)	*5 (71,42)*	*2 (28,57)*	*10 (66,66)*	*6 (40)*	*6 (37,5)*
Downregulated (%)	**2 (28,57)**	**5 (71,42)**	**5 (33,33)**	**9 (60)**	**10 (62,5)**
Total (%)	2 (28,57)	2 (28,57)	4 (26,6)	6 (40)	6 (37,5)

Differentiation markers	2 (28,57)	2 (28,57)	4 (26,6)	6 (40)	6 (37,5)
MSCs associated markers (%)	3 (42,8)	4 (57,1)	6 (40)	5 (33,33)	5 (31,2)
MSCs specific markers (%)	2 (28,57)	1 (14,28)	3 (20)	4 (26,6)	5 (31,2)
Stemness markers (%)	/	/	2 (13,3)	/	/

CS = comparison set, Y = young donor, O = old donor, and P = passage; numbers in italic and bold indicate fold regulation value.

## Abbreviations

cASCs: Canine adipose derived stem cells
MSCs: Mesenchymal stem cells
ISCT: International Society for Cell Therapy
PBS: Phosphate-buffered saline
FBS: Foetal bovine serum
DMEM: Dulbecco's Modified Eagle Medium
DMSO: Dimethyl sulfoxide
RT: Room temperature
CD: Cluster of differentiation
Ig: Immunoglobulin
APC: Allophycocyanin
PE: Phycoerythrin
FITC: Fluorescein isothiocyanate
PE-Cy7: R-Phycoerythrin-Cyanine 7
P: Passage
CS: Comparison set
Y: Young donor
O: Old donor.
